# Unexpected population fragmentation in an endangered seabird: the case of the Peruvian diving-petrel

**DOI:** 10.1038/s41598-019-38682-9

**Published:** 2019-02-14

**Authors:** Robin Cristofari, Paula Plaza, Claudia E. Fernández, Emiliano Trucchi, Nicolas Gouin, Céline Le Bohec, Carlos Zavalaga, Joanna Alfaro-Shigueto, Guillermo Luna-Jorquera

**Affiliations:** 1 Department of Biology, University of Turku, 20014 Turun Yliopisto, Turku, Finland; 2 Université de Strasbourg, Centre National de la Recherche Scientifique (CNRS), Institut Pluridisciplinaire Hubert Curien (IPHC) UMR 7178, F-67000 Strasbourg, France; 3 Laboratoire International Associé (LIA-647 BioSensib, CSM-CNRS-Unistra), 8 Quai Antoine 1er, Monaco, 98000 Monaco; 4 Centre for Ecological and Evolutionary Synthesis (CEES), Department of Biosciences, University of Oslo, Postboks 1066, Blindern, Oslo Norway; 5 Millennium Nucleus for Ecology and Sustainable Management of Oceanic Island (ESMOI), Departamento de Biología Marina, Facultad de Ciencias del Mar, Universidad Católica del Norte, Larrondo 1281, Coquimbo, Chile; 6 Department of Botany and Biodiversity Research, University of Vienna, Rennweg 14 A-1030, Vienna, Austria; 7 Department of Life Sciences and Biotechnology, University of Ferrara, 44121 Ferrara, Italy; 8 Instituto de Investigación Multidisciplinar en Ciencia y Tecnología, Universidad de La Serena, Av. Raul Bitran Nachary, La Serena, Chile; 9 Centro de Estudios Avanzados en Zonas Áridas (CEAZA), Larrondo 1281, Coquimbo, Chile; 10 Centre Scientifique de Monaco - Département de Biologie Polaire, 8, quai Antoine 1er, MC, 98000 Monaco; 11 Universidad Científica del Sur, Lima, Antigua Panamericana Sur Km 19, Lima, Peru

## Abstract

In less than one century, the once-abundant Peruvian diving petrel has become the first endangered seabird of the Humboldt Current System (HCS). This small endemic petrel of the South American Pacific coast is now an important indicator of ongoing habitat loss and of the success of local conservation policies in the HCS - an ecoregion designated as a priority for the conservation of global biodiversity. Yet so far, poorly understood life history traits such as philopatry or dispersal ability may strongly influence the species’ response to ecosystem changes, but also our capacity to assess and interpret this response. To address this question, we explore the range-wide population structure of the Peruvian diving petrel, and show that this small seabird exhibits extreme philopatric behavior at the island level. Mitochondrial DNA sequences and genome-wide SNP data reveal significant isolation and low migration at very short distances, and provide strong evidence for questioning the alleged recovery in the Peruvian and Chilean populations of this species. Importantly, the full demographic independence between colonies makes local population rescue through migration unlikely. As a consequence, the Peruvian diving petrel appears to be particularly vulnerable to ongoing anthropogenic pressure. By excluding immigration as a major factor of demographic recovery, our results highlight the unambiguously positive impact of local conservation measures on breeding populations; yet at the same time they also cast doubt on alleged range-wide positive population trends. Overall, the protection of independent breeding colonies, and not only of the species as a whole, remains a major element in the conservation strategy for endemic seabirds. Finally, we underline the importance of considering the philopatric behavior and demographic independence of breeding populations, even at very fine spatial scales, in spatial planning for marine coastal areas.

## Introduction

The large-scale oceanographic processes that structure the southern Pacific Ocean also fuel the upwelling of nutrient-rich subsurface waters along the West coast of South America^1^, resulting in one of the most highly productive oceanic systems on the planet: the Humboldt Current System (HCS)^2,3^. Local cape and island topology increases and stabilizes the upwelling activity, and generates productivity and biodiversity hotspots^4^. Together with the high endemism of its coastal ecosystems^5–7^, this makes the HCS an ecoregion of global interest for the conservation of marine biodiversity^8,9^. Concurrently, its exposure to direct threats^2^ and strong sensitivity to climate change^3,10^ make reliable information about ongoing biological processes a vital necessity. The use of seabirds as important integrative bioindicator species has been regularly stressed in this context: as upper predators, they respond both to overall ecosystem productivity and to trophic network restructuration^11–13^. However, current knowledge of HCS seabird populations is limited: demographic surveys are often patchy and lack a consistent methodological framework, resulting in over- or underestimation of population trends^14,15^.

One difficulty in studying seabird populations is their dependence on both marine and terrestrial ecosystems^16–18^, which makes them sensitive to changes on both sides of the shoreline. At sea, threats involve direct impacts of fisheries such as competition for food^13,19–23^ and bycatch^24,25^, and indirect impacts of human activities such as climate change^19,26,27^. On land, the disruption of breeding habitats can be caused by the spread of diseases^28^, sudden habitat disappearance^29^ and/or growing interaction with terrestrial predators - including humans^30^. Unlike the changes in the marine environment, disturbances on land may have local effects; failure to clearly assess the scale at which these effects occur can lead us to confuse local effects with general trends^12,31,32^, which may result in flawed conservation strategies.

**Figure 1:**
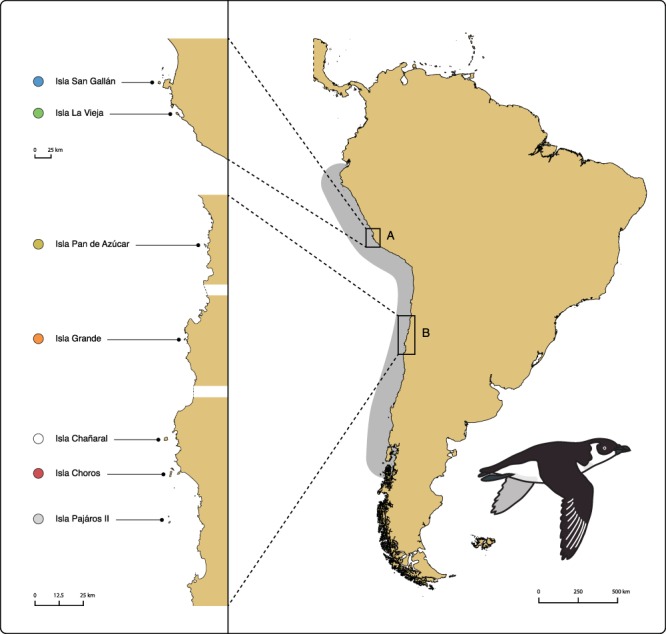
Current breeding range of the Peruvian diving petrel. (**A**) Peruvian and (**B**) chilean range of the species. On the left, close-up of the range of the species (dashed lines indicate discontinuity in our representation of the shoreline). The grey area represents the historical range of the species. Isla Chañaral is currently unoccupied, and Isla Pájaros II, in grey, could not be sampled for this study. Islands sampled for the study (Choros, Grande, Pan de Azúcar, La Vieja and San Gallán) are indicated by colored circles.

The Peruvian diving petrel (*Pelecanoides garnotii*), a small seabird endemic to the HCS, is a dramatic example of the way multiple direct and indirect threats may converge on a single species. This small insular petrel was once one of the most abundant seabirds of the HCS. In the early 20th century, it was documented from 6°S (in Isla Lobos de Tierra, Peru) to 42°S (in Isla Chiloé, Chile)^33,34^. Yet the Peruvian diving petrel was driven to near-extinction by human-induced threats that included habitat destruction through guano extraction^34–36^, poaching of adults and egg collection^35^, bycatch^35,37^, human-introduced predators^15,38^ and habitat competitors^39^, food competition with fisheries^35,40^, light pollution (Sea Shepherd Chile, pers. com.), and the effects of ongoing climate change on the productivity of the HCS^3^. As a consequence of these multiple threats, only seven nesting sites remain active in the previous 35° latitudinal range of the species^36,38,41,42^. The species’ breeding range has been fragmented into two main areas, separated by 1,300 km (Fig. 1) — one in central Peru, between 8°S and 14°S (Isla Corcovado with a handful breeding pairs, Isla San Gallán and Isla La Vieja with 12,000–13,000 pairs in total^40,43,44^), and one in northern Chile between 26°S and 29°S (Islas Pan de Azúcar, with an estimated ~220 pairs, Isla Choros, with 9,516 pairs, Isla Grande, with ~200 pairs, and Isla Pájaros II, with ~120 pairs^15,45–47^). Historical population sizes are unknown, but available information suggests a rapid decline. A striking example can be found in Isla Chañaral, in northern Chile, where ~100,000 pairs bred in 1938^38^: this population is now extinct^15^.

In contrast to this global decline, however, marginal range re-expansion and local population growth have been suggested^36^. In the past decades, small breeding groups have been discovered on the Peruvian Corcovado Island^43^, and non-breeding individuals have been sighted off northern Peru^36,42,48^. Rapid population growth was observed on La Vieja Island, Peru^44^, as well as on some Chilean islands^15,49^. However, the processes underlying these possible population recovery events are poorly understood, and it is yet unknown whether the remaining populations have effectively gone through a bottleneck, as suggested by their severe decline. Most importantly, the scarcity of knowledge on the species’ life history, phenology, and at-sea distribution casts serious doubts on the few existing surveys. It cannot be excluded that active breeding locations have only been visited by observers outside of the breeding peak, or that visits have been too few to assess population trends. Distinguishing recovery from increasing knowledge about a previously underestimated population is not a trivial problem^14^. As an example, recent census estimates for Isla Pan de Azúcar vary between ~220^45^, ~600^50^ and ~3,300 breeding pairs^51^ based on a similar methodology, without any decisive argument to explain these discrepancies. It may be that the species did not go through a population bottleneck at all, and that its resilience to habitat disruption has been much higher than previously thought.

The effects of local demographic processes in the Peruvian diving-petrel may be further blurred by the lack of genetic structure generally observed in HCS seabirds. Indeed, while most studies conducted in seabirds globally have emphasized the importance of genetic or phylogeographic structure in species dynamics^52,53^, the HCS stands out as an exception: to date, surveys have revealed a lack of genetic structure at the continental scale (e.g. Peruvian booby *Sula variegata*^54^, or Peruvian pelican *Pelecanus thagus*^55^), or very high gene flow between colonies (Humboldt penguin *Spheniscus humboldti*^56^). Promotion of high dispersal by foraging concentrations around localized upwellings^55^ has been proposed as a central underlying mechanism. If the Peruvian diving petrel fits into the same panmictic picture, demographic independence of colonies may not be assumed. In that case, observed local population growth should be interpreted as the result of complex processes, and only the range-wide assessment may be informative as to the status of the species. If, on the other hand, high philopatry and low dispersal are the norm, we can expect demographic independence between breeding locations. In that case, population trends observed at single locations may reflect actual local demographic responses (e.g. local conservation measures such as reduced guano extraction in several protected islands, and the creation of marine protected areas), rather than range-wide and species-scale processes. In addition, local gene pools should be informative about the recent demographic events at each location. Here, we use a combination of range-wide mitochondrial data and genome-wide genetic markers to (a) estimate the importance of philopatric behavior and demographic independence in the Peruvian diving-petrel, and (b) understand recent population history and its implications for the future of the species. In particular, we examine whether the current distribution of the species is better explained by local extirpation and recolonization, or rather by unexpected resilience.

## Material and Methods

### Ethics statement

This study was carried out in accordance with the recommendations of the Corporación Nacional Forestal de Chile (permit N°38/2012), the Servicio Agrícola y Ganadero de Chile (Resolution N° 7238/2013), and the Servicio Nacional de Áreas Naturales Protegidas por el Estado, Ministerio del Ambiente de Perú (permit N° 22-2018-SERNANP - RNP/J). The protocol was approved by the Bioethics Committee of the Universidad Católica del Norte, Coquimbo, Chile.

### Sample collection and DNA extraction

Blood sampling of Peruvian diving petrels was performed between April and May of 2012, outside of the reproductive peak season to minimize disturbance. A total of 109 individuals, representing seven colonies of the Peruvian diving petrel, were sampled along its breeding range (Fig. 1): two in Isla Choros, Chile (29°16′S 71°32′W, CHA and CHB, 32 and 11 individuals, respectively), one in Isla Grande de Atacama, Chile (27°15′S 70°59′W, IGA, 7 individuals), two in Isla Pan de Azúcar, Chile (26°09′S 70°41′W, AZA and AZB: 27 and 11 individuals, respectively), one on Isla La Vieja, Peru (14°15′S 76°12′W, ILV, 12 individuals), and one on Isla San Gallán, Peru (13°50′S 76°26′W, ISG, 9 individuals). Adults were captured using a mist net placed at the entrance of the colony at dusk: capturing adults as they returned to their burrows minimized the chance of sampling prospecting individuals (which presumably do not excavate a burrow until they settle for breeding). Blood was sampled from the retia in the interdigital membrane of the foot, using a heparinized microcapillary, dried on a Whatman filter paper and stored at −20 °C. DNA extraction was performed using a standard spin-column protocol (Qiagen DNeasy® Blood and Tissue kit) with minor modifications. All collected samples (N = 109) were sequenced for a fragment of the mitochondrial cytochrome-b gene. A subset of 21 samples from two islands (Isla Choros: CHA = 6, CHB = 5, and Isla Pan de Azúcar: AZA = 5, AZB = 5) was selected for single-digest RAD sequencing. We randomly selected these samples from a subset for which DNA molecular weight was above 10,000 bp (as assessed visually on a 1% agarose gel), and total available DNA quantity was above 500 ng (the starting quantity for our RAD sequencing library preparation protocol^57^).

### Mitochondrial marker sequencing

A 900 bp-long fragment of the mitochondrial cytochrome-b gene was amplified for all samples using either two or four specific primers designed from the sequence published by Nunn and Stanley^58^: as 5′-3′, yun1f: GCCCCAAACCTCCGAAAATCCCA and yun2r: GGTGATGGAGGCTAGTTGGCCG or with internal primers yun1r: GCCTGATTCGTGAAGGAAGGTGAGG and yun2f: CCACCCTAACCCGATTCTTCGCC. Amplification and sequencing initially used the four primer design, but it appeared that using only two primers yielded equally good sequences, and that design was preferred for the latest sequences (Isla Grande de Atacama, Isla San Gallán and Isla La Vieja). PCR were performed using MasterMix® (Qiagen) premixed TAQ-polymerase, dNTPs and MgCl_2_ in a total reaction volume of 12.5 μL (6.75 μL MasterMix, 2 μL of each primer pair, 0.5 μL DNA, H_2_O to a final volume of 12.5 μL). Amplification used the following conditions: 5′ at 94 °C, followed by 35 cycles of 30″ at 94 °C, 30″ at 57 °C and 1′ at 72 °C, and finally 5′ at 72 °C. PCR products were then purified using Illustra™ ExoStar™, and Sanger sequencing was performed at the ABI lab of the University of Oslo, on an ABI PRISM® 3733 analyzer. We used Geneious® v6.1.2 to process the reads: we performed visual assessment of read quality, then trimmed both ends of each read to keep only bases with an error probability below 2.5%. Finally, sequences were assembled, and a manual control was performed on the consensus sequence. All sequences were aligned and trimmed to keep only the region sequenced successfully in all samples.

### RAD library preparation and SNP typing

SNP discovery and sequencing was performed on a subset of individuals (Isla Choros: CHA = 6, CHB = 5, and Isla Pan de Azúcar: AZA = 5, AZB = 5) following a single-digest RAD-sequencing protocol^57^, modified as detailed by Cristofari *et al*.^32^ and sequenced on a half Illumina HiSeq2500 lane (V3 chemistry) at the Norwegian Sequencing Center, using paired-end, 2 × 95 bp reads. As no reference genome was available for the Peruvian diving petrel or any closely related species, data processing followed a two-step protocol. First, loci were built *de novo*, using the Stacks v1.28 pipeline^59,60^, and a synthetic reference RADome was built – an approach suggested by Hoffman *et al*.^61^ and that allows the use of well-established SNP calling algorithms in the absence of a closely-related species’ reference genome, while avoiding biases towards slowly-evolving regions due to differential success of alignment. Second, this reference RADome was used to perform alignment-based SNP typing. The workflow was as follows: (a) sequence de-multiplexing and *de novo* locus assembly was done according to in-line barcodes using Stacks’ *process_radtags.pl* script and *denovo_map.pl* pipeline, with a minimum depth of 3X, and a maximum of 5 mismatches allowed between alleles at a single locus (both within and between individuals). This level is, in our experience, reasonable for seabirds: it is the “convergence level” in King penguins, according to Paris *et al*.^62^, and indeed, changes in parameters within the 3–6 range only marginally affected our assembly (both in terms of total number of loci, and number of polymorphic loci). The corresponding paired-end contigs were assembled using Velvet^63^ and Stacks, setting a minimum contig length of 200 bp to filter out multiple non-overlapping short paired-end reads. (b) The resulting loci were concatenated together with their respective paired-end contigs, separated by a padding of 500 ‘N’s, to form the scaffolds of a reduced reference genome, or RADome^61^. At this point, loci comprising multiple paired-end contigs (a potential sign of collapsed paralogous loci) were removed. The raw paired-end fastq files were then mapped onto this RADome using Bowtie2^64^, with paired-end, concordant mapping only, a maximum distance between both reads of 500 bp (corresponding to our fxed-length N-padding), and otherwise default settings. The resulting alignments were filtered using Samtools 0.1.19^65^, PicardTools 1.113 (http://picard.sorceforge.net), and custom R and shell scripts: we discarded orphaned reads and low-quality pairs (a minimum Phred-scaled quality score of 30 was used), and further filtered the remaining aligned reads to remove PCR and optical duplicates. (c) SNP and genotype calling was restricted to the first-in-pair reads. We then used GATK’s HaplotypeCaller pipeline^66^ to call SNPs and genotypes simultaneously in all samples, using first reads only, with standard parameters except for population heterozygosity which was set to 0.01, and retaining only di-allelic, non-indel sites sequenced in at least 75% of samples. We retained only 1 SNP per locus (arbitrarily set as the first SNP in read order). We repeated key analyses using ANGSD 0.900^67,68^ to estimate allele frequency and genotype likelihoods, without hard-calling genotypes, an approach that has been shown to be much more robust for low-coverage data^68,69^.

### Analysis of population structure

Summary statistics (nucleotide diversity π and Tajima’s D) were calculated using DnaSP^70^ for mtDNA, and adegenet^71^ for SNP data. Analysis of molecular variance was performed for both datasets using Arlequin v3.5^72^, in a locus-by-locus format for SNP data, and haplotypic format for mtDNA. We performed 15,000 permutations to assess significance levels. Additionally, significance level for pairwise Φ_ST_ estimates from mtDNA were corrected using the Benjamini-Yekutieli procedure^73^ using the R package *multtest*^74^. On the other hand, pairwise SNP-based comparisons were hierarchical (one pairwise comparison within each island, and one pairwise comparison between the two sampled islands), and each was considered to test for an independent null hypothesis, so that no correction was applied. A neighbor net was built in SplitsTree^75^ either directly from the mtDNA sequences, or from a matrix of pairwise Hamming distances calculated in PLINK v1.9^76^ from SNP data. Additionally, a maximum likelihood tree was built from the cytochrome-b sequences in RAxML^77^, using a simple HKY + G substitution model as supported by the Akaike Information Criterion (AIC)-based model selection performed in *JModelTest2*^78^, and otherwise default parameters. This tree was used to build a Fitch-distance haplotype network using Fitchi^79^.

Principal components analysis was performed on genome-wide SNP data either using called genotypes in *adegenet*, or raw genotype likelihoods in ngsTools^80^. Admixture between populations was estimated using two different approaches: (1) a classical model-based clustering approach relying on the hypothesis of Hardy-Weinberg equilibrium (HWE)^81^, as implemented with an empirical Bayes algorithm either in fastStructure^82^ for called genotypes, or in ngsAdmix^83^ for raw genotype likelihoods, retaining only sites that did not violate HWE, with a number of components ranging from 1 to 4, and 100 bootstrap replicates. Model complexity was chosen using Evanno’s ΔK method^84^. (2) In order to assess the impact of the HWE assumption, we used a “model-free” approach implemented in the *adegenet* pca-coordinate k-means clustering algorithm^85^.

### Model-based estimation of population history

We relied on two separate coalescent-based approaches to estimate the amount of gene flow between the northern and southern ends of the species’ Chilean range (i.e. between Isla Choros and Isla Pan de Azúcar) based on genome-wide SNP data. First, population sizes and migration rates were co-estimated based on multilocus short-haplotype data in a Bayesian framework, as implemented in *Migrate-n*^86^. We selected three random sets of 50 95-bp-long nuclear loci containing 4 to 6 polymorphisms each as an unbiased representation of the neutrally evolving part of the genome (a protocol described in Trucchi *et al*.^87^), phasing being provided by the Illumina read identity. In order to correct for potential allele dropout, we randomly sampled one haplotype (i.e. allele) only for each individual, at each locus, as proposed by Cristofari *et al*.^32^. We ran a cold chain and 3 heated chains under a static heating scheme, raising the cold chain to a power of 1.5, 3 and 1e6 and proposing chain swapping every 100 steps. Chains were run for 50,000,000 generations, recording every 500 generations, with a 5,000,000-generation burn-in, specifying uniform priors both for population sizes (Θ, bounded between 0 and 0.1 with a δ of 0.01), and for the migration rates (M, bounded at 4,000 with a δ of 400). Proper mixing under these conditions was ensured by using the highest parametrization model and checking convergence between the three independent random datasets. Models were ordered by log Bayes factors^88^, using the thermodynamic integration approximation of marginal likelihood ds. Four models were tested: (1) the two islands are fully isolated populations; (2) the two islands are independent populations exchanging migrants with symmetrical gene flow; (3) the two islands are independent population exchanging migrants with asymmetrical gene flow; and (4) the two islands belong to a single, panmictic population.

Co-estimation of population size, gene flow and population history was performed by explicit model testing in *fastsimcoal2*^89^, through AIC comparison of the optimized composite likelihood of the two-dimensional folded allele frequency spectrum under six different models (see input files in Supplementary information). We first tested two simple models: (1) a full isolation model with two stable, independent populations, and (2) a classical isolation-with-migration model, with population sizes, migration rates and divergence time as free parameters. We then tested four more complex scenarios (3–6), in which the two populations diverged after the last glacial maximum, ca. 7,000 generations ago (a conservative biogeographic estimate for most taxa). Under model (3) human activities have no impact on the populations, and constant gene flow is maintained between the islands until the present, with no population size changes. Under model (4) population size does not change, but gene flow is disrupted at some point during the past. Under models (5) and (6), population size changes during the past, and either gene flow remains constant (model 5) or gene flow also changes (model 6). We generated 50 nonparametric spectrum bootstrap replicates and performed 50 parallel runs for each model and each bootstrap replicate, keeping only the one with the highest composite log-likelihood. Each run required a maximum of 80 ECM cycles, simulating 100,000 spectra at each step.

Models were calibrated using the general background genomic mutation rate for water birds, 1.6 e^−3^ substitutions.site^−1^.Myr^−1^, as established from a wide panel of genomic data by Zhang *et al*.^90^, or μ = 9.6 e^−9^ substitutions.site^−1^.generation^−1^ considering a generation time of 6 years^91^. *Migrate-n* models, however, were based on RAD loci containing 4 to 6 polymorphic sites: these cannot be calibrated directly using the general background substitution rate. Considering that most of the RADome is assumed to evolve neutrally, the number of polymorphic sites per locus is expected to follow a Poisson distribution, and the background substitution rate μ applies for the expected value λ of that distribution. Therefore, after verifying the goodness-of-fit of the Poisson model we extracted the expected substitution rates for loci with S = 4, S = 5 or S = 6 SNPs, as S/(λ/μ), and used their weighted average to calibrate our reconstructions^32^.

### Range-wide process analysis from mtDNA data

In order to better understand range-wide demographic processes, we used sampling locations as the only prior hypothesis on phylogeographic processes, and inferred locations at ancestral nodes in a Bayesian framework as implemented in *BEAST2*^92,93^. We partitioned data so as to set a separate model for third-codon positions, using a simple HKY + G mutation model as inferred in *jModelTest2*^78^ with three gamma-distributed categories. Since we worked at an intraspecific level without external prior information on population history, we chose to remain within a simple constant-population coalescence model with a strict molecular clock. The sampling chain was run for 100,000,000 generations, recording every 5,000th state. This allowed the generation of sufficient effective sample sizes (ESS > 500) for all parameters in the model. Clock calibration used the estimate provided for the cytochrome-b gene by Nunn and Stanley^58^ for medium-sized Procellariiformes (the order of seabirds that includes the *Pelecanoides* diving-petrel genus), of 0.90% per million years.

## Results

### Range-wide mtDNA analysis

Analysis of cytochrome-b sequences unequivocally indicated a high degree of reproductive isolation between the main breeding locations across the range of the Peruvian diving-petrel. Pairwise Φ_ST_ values were high, and the majority deviated significantly from a random distribution of the same sequences (Table 1). This was particularly the case for longer-distance comparisons. The two neighboring Peruvian colonies of Isla La Vieja and Isla San Gallán, on the other hand, did not differ significantly. Within the islands of Choros and Pan de Azúcar, differences between colonies were insignificant. Analysis of molecular variance, with colonies gathered into four groups (a-both Choros colonies, b-Isla Grande de Atacama, c-both Pan de Azúcar colonies, and d-both Peruvian colonies) also supported significant differentiation between groups. Difference between groups accounted for 14.87% of the total variation, while difference between colonies within each group accounted for 12.11%. Differentiation between groups was significant (Φ_ST_ = 0.270, *p*-value < 0.05), and so was differentiation between colonies within groups (Φ_SC_ = 0.142, *p*-value < 0.05). All 109 pooled sequences contained 14 segregating sites and 14 different haplotypes. Despite rather asymmetric sample sizes, standard indices of molecular diversity were homogeneous (Table 2).

**Table 1:** Pairwise Φst values computed from the cytochrome-b mtDNA sequences between each Peruvian diving petrel breeding colony.

	AZA	AZB	CHA	CHB	IGA	ISG	ILV
AZA	—	0.269 ± 0.004	0.001 ± 0.000	0.000 ± 0.000	0.180 ± 0.004	0.000 ± 0.00	0.000 ± 0.00
AZB	0.015	—	0.250 ± 0.004	0.001 ± 0.000	0.734 ± 0.004	0.001 ± 0.000	0.001 ± 0.000
CHA	0.129**	0.016	—	0.012 ± 0.001	0.660 ± 0.0045	0.005 ± 0.000	0.001 ± 0.000
CHB	0.398**	0.364**	0.165	—	0.0467 ± 0.002	0.256 ± 0.0045	0.185 ± 0.004
IGA	0.046	−0.051	−0.030	0.205	—	0.048 ± 0.002	0.022 ± 0.001
ISG	0.421**	0.417**	0.209*	0.032	0.212	—	0.879 ± 0.003
ILV	0.443**	0.435**	0.227**	0.0412	0.248	−0.086	—

Below diagonal: mean Φst values. Above diagonal: *p*-values for significance as assessed against a random distribution of the same sequences, with 10,000 permutations. Significant differences from random expectations based on permutations of the same dataset are signalled by asterisks, after Benjamini-Yekutieli correction (‘*’ for a corrected p-value < = 0.05, and ‘**’ for a corrected p-value < = 0.01). AZA/AZB and CHA/CHB are the two breeding colonies of Isla Pan de Azúcar and Isla Choros, respectively, IGA is Isla Grande de Atacama, ILV is Isla La Vieja and ISG is Isla San Gallán.

**Table 2:** Summary statistics of the cytochrome-b mtDNA haplotype sequences for the Peruvian diving petrel breeding colonies.

	AZR	CHR	IGA	ILV	ISG	ALL
Haplotype diversity	H_AZR_ = 0.711	H_CHR_ = 0.632	H_IGA_ = 0.810	H_ILV_ = 0.667	H_ISG_ = 0.778	H_ALL_ = 0.765
Nucleotide diversity	π_AZR_ = 0.00125	π_CHR_ = 0.00220	π_IGA_ = 0.00204	π_ILV_ = 0.00185	π_ISG_ = 0.00203	π_ALL_ = 0.00213
Nucleotide diversity (nuclear SNP)	π_AZR_ = 0.00226	π_CHR_ = 0.00224	—	—	—	—
Pairwise differences	K_AZR_ = 0.997	K_CHR_ = 1.75	K_IGA_ = 1.62	K_ILV_ = 1.47	K_ISG_ = 1.61	K_ALL_ = 1.70
Tajima’s D	D_AZR_ = −0.649	D_CHR_ = −0.430	D_IGA_ = −1.02	D_ILV_ = 0.384	D_ISG_ = −0.673	D_ALL_ = −0.672
Tajima’s D (nuclear SNP)	D_AZR_ = −0.361	D_CHR_ = −0.364	—	—	—	—

AZR: Isla Pan de Azúcar, CHR: Isla Choros, IGA: Isla Grande de Atacama, ILV: Isla La Vieja, ISG: Isla San Gallán, ALL: pooled samples. ns: *p*-value > 0.05. Values are computed from the cytochrome B sequence, or from nuclear SNP data when specified.

**Figure 2:**
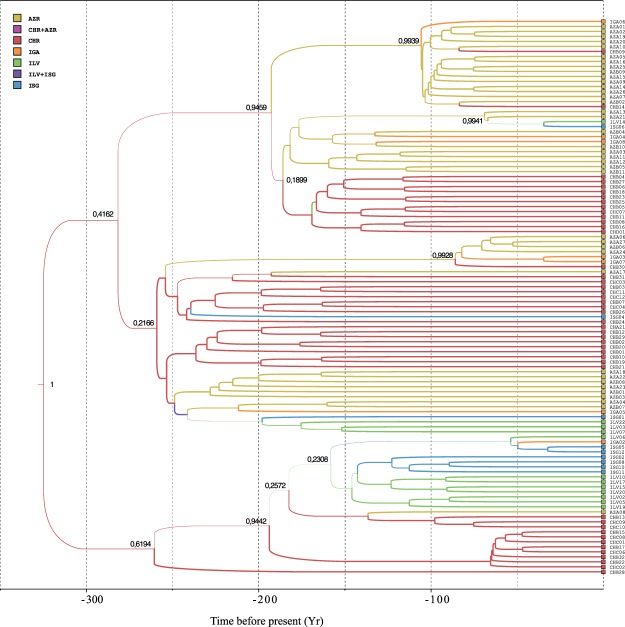
Maximum-likelihood phylogenetic tree of the Peruvian diving petrel based on cytochrome-b sequences and the HKY + G substitution model. Calibrated time tree with inferred ancestral location. Colors indicate the location, either sampled (at the tips), or inferred (along the branches). Posterior probability is indicated at the main nodes.

**Figure 3:**
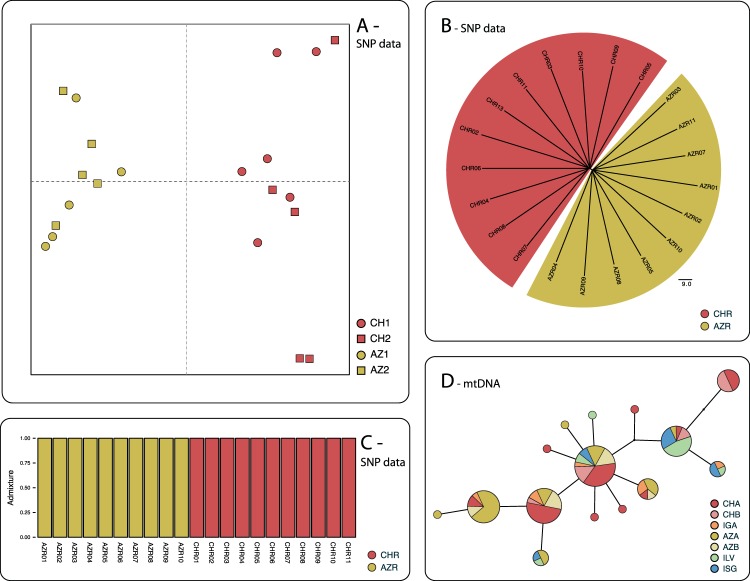
Population genetic structure of the Peruvian diving petrel. (**A**) Principal component analysis performed on genome-wide SNP data generated for the Chilean breeding colonies of Isla Choros (CH1 and CH2) and Isla Pan de Azúcar (AZ1 and AZ2), using raw genotype likelihoods calculated by ngsTools; (**B**) NeighborNet of the Isla Choros (CHR) and Isla Pan de Azúcar (AZR) samples built from nuclear SNP data; (**C**) Individual components of admixture between samples of Isla Choros (CHR) and Isla Pan de Azúcar (AZR), as inferred from genome-wide SNP genotype likelihood data using ngsAdmix (median values from 500 bootstrap replicates); (**D**) Fitch distance haplotype graph based on the mitochondrial cytochrome-b gene sequences of all the breeding colonies sampled for the study (disc area proportional to the number of haplotype copies). AZA/AZB and CHA/CHB refer to the two breeding colonies sampled in Isla Pan de Azúcar and Isla Choros, respectively, IGA to Isla Grande de Atacama, ILV to Isla La Vieja and ISG to Isla San Gallán. Color codes on all figures are as in Fig. 1.

The coalescent-based joint reconstruction of the gene tree for cytochrome-b and geographic location probability at ancestral nodes performed in *BEAST2* provided more information on the present-day structure of Peruvian diving petrel populations (Fig. 2). The highest posterior probability was for a common ancestor for all samples in Isla Choros, and subsequent colonization of the Chilean and Peruvian range from there - although a common ancestor in Isla Pan de Azúcar cannot be rejected with confidence. Using a common estimate of mitochondrial substitution rate in medium-sized Procellariiformes, this common ancestry is inferred to be rather recent - about the early 17^th^ century. Most of our samples from Peru seem to stem from a single migration event, although a few others are inferred to be more recent migrants from Isla Choros and Isla Pan de Azúcar. Most remarkably, samples from Isla Grande de Atacama appear to result from multiple independent migration events, with sources in Choros, Pan de Azúcar and Peru. These observations are consistent with the maximum-likelihood-tree-based Fitch network (Fig. 3D).

### A focus on two islands using genome-wide SNP data

We found genome-wide short-locus RAD-sequencing and mitochondrial data to be in good agreement. The Illumina sequencing lane yielded 182,319,948 paired-end reads with a mean phred-scaled quality score of 37, of which 144,048,478 were retained after barcode and cut-site sequence filtering. The final database included 65,582 distinct RAD loci, of which 37,170 were polymorphic, with an average of 1.3 SNPs per locus. After PCR-duplicate removal, SNP calling from the higher-quality reads in GATK yielded 43,175 SNPs, of which 32,964 were genotyped in at least 75% of individuals, with a mean depth of 3.2X and a median depth of 3.1X. Nucleotide diversity computed from genome-wide SNP data was very similar across islands (see Table 2 - and pooled diversity π = 0.00233), in perfect agreement with mtDNA-based values (see Table 2), and at a level in accordance with the expectation for long-lived seabirds (Romiguier *et al*. 2014). Here again, Tajima’s D supports neutral evolution at the variable sites (see Table 2). Small sample sizes have been shown to bias genome-wide estimates of population size in cases of non-neutral sequence evolution, or exponential population growth^94,95^. Neither appear to be the case in our data, although strictly quantitative interpretation of the demography is of course impossible.

All analyses support a high level of genetic divergence at such a short geographic distance for seabirds^52–56^. The fixation index was high for genome-wide SNP data (Fst = 0.049 ± 0.008, averaged over 500 SNP windows). Analysis of molecular variance also supports significant differentiation between Isla Choros and Isla Pan de Azúcar. Based on SNP data, the difference between islands accounts for 2.51% of the total variation, while differences between colonies within each island are virtually nonexistent (−0.099% per AMOVA). Genetic differentiation between island groups is significant (Φ_ST_ = 0.025, *p*-values < 0.05), but differentiation between colonies within islands is not (Φ_SC_ = −0.001, *p*-value = 0.795). Principal component analysis of the SNP data clearly supports a two-population structure (Fig. 3A) Although the first component only accounts for 6.62% of the total variance, it discriminates widely between individuals from Isla Choros and Isla Pan de Azúcar - while the second and following components only summarize inter-individual variance. The idea of a low-level but highly consistent separation is also supported by a Hamming-distance-based neighbor net (Fig. 3B); terminal edges are much longer than structural inner edges, yet sorting is complete between the two islands (a structure that is however rather poorly supported by a mtDNA haplotype network, see Fig. 3D). Finally, clustering analyses, whether based on genotype likelihood (Fig. 3C) or on genotype calls, strongly support a two-population model, over both panmixia and higher complexity models. It should be noted, however, that our sampling design does not allow us to positively differentiate a two-population model from the discrete sampling of an isolation-by-distance gradient, that may have formerly included the intermediate (but extinct) Isla Chañaral population, as well as the current Isla Grande de Atacama population.

### Isolation and migration analysis

*Migrate-n* models of connectivity support a structure with low migration between separated populations. Bayes factor (BF) model comparison across three independent replicates allows unambiguous rejection of both the panmixia and full isolation hypotheses. BF did not allow a clear choice between symmetrical and asymmetrical migrations, although since BF does not take into account the number of estimated parameters, the lower-parameterization symmetrical migration model should be preferred. Under this model, Θ_CHR_ = 0.00697 ± 8.0829e^−5^, Θ_AZR_ = 0.00747 ± 0.000212 and M = 2075 ± 10.6. Using the reconstructed substitution rate for highly polymorphic RAD loci μ = 3.55.e^−8^ substitutions site^−1^.generation^−1^, inferred effective population sizes scale to N_CHR_ ≈ 58,600 and N_AZR_ ≈ 62,800, with a symmetric migration rate of M ≈ 3% effective migrants per generation.

**Figure 4:**
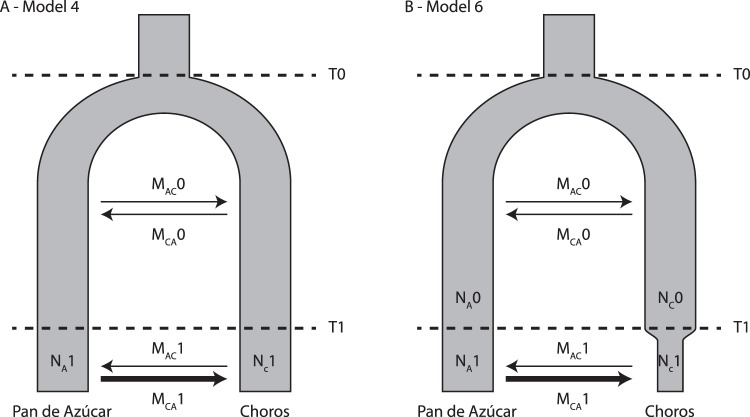
Selected *fastsimcoal2* models of demographic history. (**A**) Model 4 infers a change in the rate of migration from Isla Pan de Azúcar to Isla Choros in a recent past, and (**B**) model 6 infers change in both migration rate from Isla Pan de Azúcar to Isla Choros, and in population size at Isla Choros. *Parameter names and values are detailed in* Table 3.

Joint minor-allele frequency spectrum-based inferences (as performed in *fastsimcoal2*) were globally consistent with this model, both in terms of population sizes and migration rates, which fall within the same orders of magnitude. Parameter values and confidence intervals are given in Table 3. AIC supports a model with a recent change in the migration regime (about 200 generations ago), but no population size change (our fourth model, see Fig. 4A). Population sizes are widely asymmetric despite equal prior specification. Migration from Choros to Pan de Azúcar is inferred stable, but increases at least ten-fold from Pan de Azúcar to Choros. The second-best AIC score supports a model with changes in migration regime and in population size (model 6, see Fig. 4B): inferred migration patterns were similar, although change occurs at a slightly more recent time. Population size in Pan de Azúcar was stable or increased slightly, but decreased in Choros.

**Table 3:** Parameter estimates from *fastsimcoal2* demographic reconstructions.

	Model 4		Model 6	
	Median	CI95%	Median	CI95%
T0	(7,000)		(7,000)	
M_AC_0	2.756e-4	[2.397e-4; 3.025e-4]	2.853e-4	[2.466e-4; 3.128e-4]
MCA0	1.146e-5	[1.121e-5; 1.209e-5]	1.135e-5	[1.018e-5; 1.207e-5]
N_A_0	—	—	1,014	[1,010; 1,019]
N_C_0	—	—	95,542	[88,815; 101,124]
T1	207	[82; 375]	75	[6; 231]
M_AC_1	4.268e-3	[2.553e-3; 1.041e-2]	1.103e-2	[3.724e-3; 1.116e-1]
M_CA_1	1.170e-5	[1.124e-5; 1.240e-5]	1.224e-5	[1.122e-5; 2.226e-5]
N_A_1	1,013	[1,010; 1,018]	1,027	[1,011; 17,702]
N_C_1	91,35	[87,977; 95,509]	23,832	[10,068; 36,104]

Model structures are illustrated in Fig. 4. Times are given in generations, population sizes in effective individuals, and migration rates are mutation-scaled. Parameter names are as follows: T0 is the fixed divergence, set at 7,000 generations. T1 is the time of regime change. M_AC_0, M_CA_0, M_AC_1 and M_CA_1 are the migration rates from Pan de Azúcar to Choros and from Choros to Pan de Azúcar, during period 0 and period 1, respectively. For model 4, N_A_1 and N_C_1 are the (constant) population sizes in Pan de Azúcar and Choros. For model 6, N_A_0, N_C_0, N_A_1 and N_C_1 are the population sizes in Pan de Azúcar and Choros, during period 0 and period 1, respectively.

## Discussion

### A highly structured population

The range-wide colonies of the Peruvian diving petrel stand out as remarkably structured, despite short geographic distances and a tumultuous recent history. Our study brings strong support to the idea of distinctive populations in each island group and limited gene flow between them. Isla Grande de Atacama, however, stands out as an exception: the small population seems mostly a sink for migrant birds from all other populations, and may stem from a recent re-colonization event. Genome-wide SNP-based analyses are in full accordance with mtDNA sequence analyses, and support a complete sorting of individuals between Isla Choros and Isla Pan de Azúcar in Chile, albeit with moderate differentiation.

The good agreement between nuclear and mitochondrial signals in the Isla Choros - Isla Pan de Azúcar comparison allows us to rely confidently on mtDNA sequence data alone for range-wide analysis. Some aspects, however, deserve consideration. The clearer sorting of individuals observed with nuclear data compared to mtDNA (see e.g. Fig. 3B *vs*. Fig. 3D) is most likely a consequence of the much higher resolution of the large neutral nuclear marker dataset compared to the relatively short and non-recombining functional cytochrome-b gene sequence. Sex-specific dispersal has often been proposed as an explanation in cases of discrepancies between nuclear and mitochondrial signals. However, this interpretation has been questioned^96^, and the difference in coalescence rate of haploid and diploid markers has been convincingly proposed instead. In our case, the much larger sample size used for mtDNA analysis (83 individuals for Choros-Azúcar comparison) compared to nuclear analysis (21 individuals) may also account for a large part of this apparent difference. The resolution and the strength of the signal, however, should not be confused: the weaker, but clearer nuclear signal may characterize the true isolation processes more accurately than the larger and more contrasted mitochondrial dataset^94^.

Reconstructed population sizes and gene flow between Isla Choros and Isla Pan de Azúcar are globally consistent between haplotype-based and spectrum-based approaches, although with considerably higher precision for the spectrum-based inferences. Both *Migrate-n* and *fastsimcoal2* models allow us to reject panmixia as well as full isolation between colonies, and support partial isolation, with ongoing gene flow. However, the models differ in two important points. First, *Migrate-n* suggests equal population sizes, with Ne ≈ 60,000 for each population, while *fastsimcoal2* infers unequal (yet stable) population sizes, with N_CHR_ ≈ 90,000 and N_AZR_ ≈ 1,000. Despite these contrasting results, however, both models propose an effective population size that is considerably higher than the present-day census: a total population of ~90,000 to ~120,000 effective breeders is in clear opposition to the currently observed population of ~19,000 breeders in Isla Choros^47^ and ~4400 in Isla Pan de Azúcar^45^, but more in tune with the historical populations (e.g. 100,000 breeders on the major breeding location of Isla Chañaral in 1938, and similarly large colonies elsewhere^38^)—a direct testimony to the rapidity and extent of the population collapse in the past decades^97^. Second, *Migrate-n* inferred symmetric and relatively high gene flow (~3% per generation), whereas *fastsimcoal2* supports much lower, asymmetric gene flow: northward migration (from Isla Choros to Isla Pan de Azúcar) is stable and very low (~0.001% per generation), while southward migration is higher, and increases from ~0.03% to ~0.4% in the recent past. Thus the haplotype-based reconstruction generally suggests a “homogenized” system, with population sizes averaged by higher gene flow. In contrast, the spectrum-based approach offers a more nuanced reconstruction that better reflects the observed state of the populations. The higher resolution of the spectrum-based approach, however, is expected. First, it is able to use the information from the full RADome, and not only from a random subset of loci. Second, haplotype-based methods chiefly capture events occurring at a substitution (i.e. millennial) scales, while spectrum-based approaches also capture drift in allele frequencies that may occur in a few generations, thus making them more suitable for the recovery of recent population history. Therefore, we may more appropriately consider *Migrate-n* reconstructions as a general framework that does not contradict the more precise reconstruction given by our *fastsimcoal2* model.

### A northward colonization from Chile

Our results allow us to understand better the recent history of the species in its entire range. First, the large divergent gene pool of Isla Pan de Azúcar excludes the idea of a local extinction or near-extinction followed by gradual re-colonization on that island. Indeed, local divergence on Isla Pan de Azúcar is comparable to that of the “refugial” Isla Choros population, and models proposing more recent colonization or steep population growth in the former are systematically rejected. Thus our data strongly support the idea that the Peruvian diving petrel was, in fact, always remnant in Isla Pan de Azúcar, despite having been overlooked by several successive surveys, probably made outside the peak breeding activity season. Our data also suggest that the minimum population size in Isla Pan de Azúcar never went under a critical threshold, since no loss of diversity appears in our data compared to historically more stable populations (e.g. Isla Choros or the Peruvian islands). If indeed the present-day population is growing (as suggested by the comparison of recent studies^45,50,51^) and not just stagnating, it is receiving very few migrants from other colonies in the process. This is in contrast to the small colony of Isla Grande de Atacama, which seems to be entirely made up of migrants from nearby colonies, and may well have gone extinct at some point in the century - it was *de facto* first identified in 2000^15^. Finally, although fueled by immigration from Chile in the past, the Peruvian colonies clearly exhibit signs consistent with the founder effect, with most samples from Isla La Vieja and Isla San Gallán stemming back to a single common ancestral haplotype.

### Conservation actions must be island-based

The observed level of population isolation has important implications for the conservation of the Peruvian diving petrel. While it has been suggested that the panmictic character of seabird populations (such as the HCS populations of Peruvian pelican or Peruvian booby) increases their robustness in the face of environmental change^32,55^, the high fragmentation of the Peruvian diving petrel population, on the other hand, should add to its already great vulnerability to land and marine habitat destruction. Indeed, high philopatry and low dispersal imply that the loss of a safe breeding island associated with a productive foraging area will lead to a local extinction and irremediable loss of genetic diversity.

An associated prediction is that current populations are demographically independent^98^. The observed local trajectories are unlikely to be an artifact of changing migratory fluxes: they should represent the true local demographic trend. However, this observation is double-edged. In an increasingly well-monitored population such as the one in Isla Choros, the local census increase^47^ can confidently be interpreted as the positive outcome of successful conservation measures - in particular the creation of the Humboldt Penguin National Reserve (that includes Isla Choros and the surrounding marine area). This is a strong encouragement to persevere in such endeavors. However, this demographic independence leaves us with serious doubts when censuses are inconsistent over time, as is the case in Isla Pan de Azúcar, as we cannot account for large and rapid population size changes in such a long-lived species without inferring important migratory flux. A conservative interpretation of census data is necessary in this case, and we should consider that faulty or mistimed surveys better account for the observed census changes^14^. Thus the population increase in Pan de Azúcar, and probably also in the Peruvian islands, should be interpreted carefully, and should not lead us to reconsider the species’ status until more reliable time-series data are available.

Most importantly, our results make it especially clear that any conservation strategy for the Peruvian diving petrel in the HCS should focus on each island and the surrounding marine areas^7^ as a significant conservation unit. Within islands, colonies do not stand out as genetically separate entities, although philopatric behavior may still apply at that scale for stretches of a few generations. But the distinctness of each island’s gene pool, as well as their inferred demographic independence, makes the conservation of each of the seven remaining breeding islands an absolute necessity for the survival of this once extremely abundant and now endangered seabird species. Indeed, despite the positive trend observed in the last decade on Isla Choros, important threats remain both for the nesting sites and for the marine habitats of diving petrels. One of the four islands where petrels nest (Isla Pájaros II, currently with no legal protection) is still under a valid permit of guano extraction, and ongoing extraction of guano was confirmed on the island as recently as 2003^15^. Future plans for mining projects include building two large harbors (http://www.conocedominga.cl, and http://www.capmineria.cl/proyecto-puerto-cruz-grande/) near the central breeding location of the specie, Isla Choros. This could affect significantly both the coastal foraging areas of the specie (through increased marine transit) and nesting areas (through direct effects of increased human intrusion), and could also increase mortality induced by lights. Our results clearly show that the conservation of every breeding location is essential for preserving the full remaining genetic diversity of this already severely reduced specie, and maximizing the chances of recovery after its massive historical decline. Thus they add compelling support to previous recommendations of the need to provide formal protection to several HCS islands that harbor a biodiversity of both regional and global importance, including colonies of endemic seabirds, and that urgently need strict legal protection status^7^.

## Data Availability

All data used in present study are available from the NCBI portal. Mitochondrial sequences have reference numbers MK113717 to MK113815, while raw Illumina reads are available from the Sequence Read Archive (www.ncbi.nlm.nih.gov/sra) under the BioProject accession number PRJNA498806. Input files for *fastsimcoal2* are available from www.figshare.com, with DOI 10.6084/m9.figshare.7262138.
